# Tetrel Bonding in Anion Recognition: A First Principles Investigation

**DOI:** 10.3390/molecules27238449

**Published:** 2022-12-02

**Authors:** Pradeep R. Varadwaj

**Affiliations:** 1 Department of Chemical System Engineering, School of Engineering, The University of Tokyo 7-3-1, Tokyo 113-8656, Japan; pradeep@t.okayama-u.ac.jp or prv.aist@gmail.com; 2 Molecular Sciences Institute, School of Chemistry, University of the Witwatersrand, Johannesburg 2050, South Africa

**Keywords:** tetrel bond, non-covalent interactions, weak-to-strong interaction energy, dative bond formation, chemical bonding, anion recognition, MESP analysis, charge-density analysis, first-principles calculations

## Abstract

Twenty-five molecule–anion complex systems [I_4_Tt···X^−^] (Tt = C, Si, Ge, Sn and Pb; X = F, Cl, Br, I and At) were examined using density functional theory (ωB97X-D) and ab initio (MP2 and CCSD) methods to demonstrate the ability of the tetrel atoms in molecular entities, I_4_Tt, to recognize the halide anions when in close proximity. The tetrel bond strength for the [I_4_C···X^−^] series and [I_4_Tt···X^−^] (Tt = Si, Sn; X = I, At), was weak-to-moderate, whereas that in the remaining 16 complexes was dative tetrel bond type with very large interaction energies and short Tt···X close contact distances. The basis set superposition error corrected interaction energies calculated with the highest-level theory applied, [CCSD(T)/def2-TZVPPD], ranged from −3.0 to −112.2 kcal mol^−1^. The significant variation in interaction energies was realized as a result of different levels of tetrel bonding environment between the interacting partners at the equilibrium geometries of the complex systems. Although the ωB97X-D computed intermolecular geometries and interaction energies of a majority of the [I_4_Tt···X^−^] complexes were close to those predicted by the highest level of theory, the MP2 results were shown to be misleading for some of these systems. To provide insight into the nature of the intermolecular chemical bonding environment in the 25 molecule–anion complexes investigated, we discussed the charge-density-based topological and isosurface features that emanated from the application of the quantum theory of atoms in molecules and independent gradient model approaches, respectively.

## 1. Introduction

Ion–molecule interactions are fascinating in chemistry [1,2,3,4], biology [5], and materials science [6,7,8]. These interactions are ubiquitous in many chemical systems in solid, liquid, and gas phases and play an important role in sensing, extraction, transport, assembly, and catalysis [6]. They appear between an anion at the molecular (or atomic) level and a neutral molecule, or between a cation and a neutral molecule. The Cambridge Structural Database (CSD) [9] has cataloged many such chemical systems in the crystalline phase [10,11].

**Figure 1:**
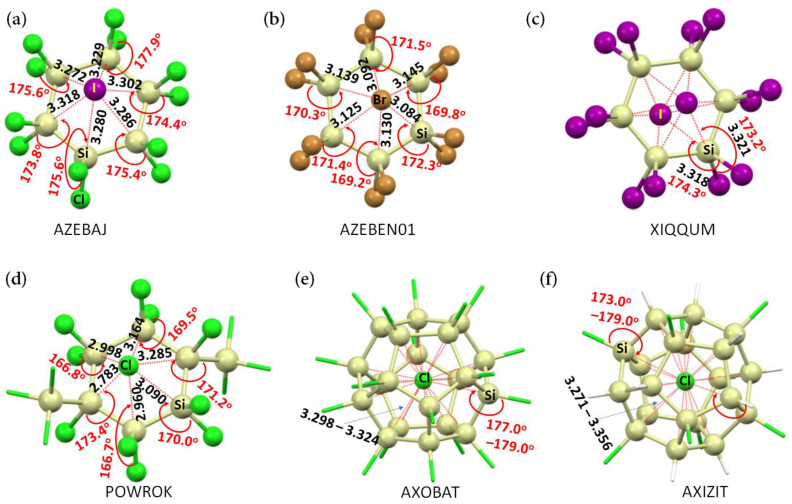
Illustration of molecule–anion interactions in the anionic part of some selected chemical systems cataloged in the CSD [9]. The crystals include: (**a**) bis(tetrabutylammonium) dodecachlorohexasilinane bis(iodide) dichloromethane solvate [2(C_16_H_36_N^+^),Cl_12_Si_6_,CH_2_Cl_2_,2(I^−^)] [12]; (**b**) bis(tetrabutylammonium) bis(bromide) dodecabromohexasilinane [2(C_16_H_36_N^+^),Br_12_Si_6_,2(Br^−^)] [13]; (**c**) bis(tetrabutylphosphanium) dodecaiodohexasilinane bis(iodide) [2(C_16_H_36_P^+^),I_12_Si_6_,2(I^−^)] [13]; (**d**) bis(tetraphenylphosphonium) 1,1,2,2,3,4,4,5,5,6-decachloro-3,6-bis(trichlorosilyl)hexasilinane bis(chloride) [2(C_24_H_20_P^+^),Cl_16_Si_8_,2(Cl^−^)] [14]; (**e**) triphenyl-N-(triphenylphosphanylidene)phosphaniminium chloride 1,2,3,4,5,6,7,8,9,10,11,12,13,14,15,16,17,18,19,20-icosachloroundecacyclo [9.9.0.0^2,9^.0^3,7^.0^4,20^.0^5,18^.0^6,16^.0^8,15^.0^10,14^.0^12,19^.0^13,17^]icosasilane chloroform solvate [C_36_H_30_NP_2_^+^,Cl_20_Si_20_,2(CHCl_3_),Cl^−^] [15]; (**f**) bis(triphenyl-N-(triphenylphosphanylidene)phosphaniminium) chloride 4-methylbenzene-1-sulfonate 1,3,5,8,10,13,16,19-octachloroundecacyclo [9.9.0.0^2,9^.0^3,7^.0^4,20^.0^5,18^.0^6,16^.0^8,15^.0^10,14^.0^12,19^.0^13,17^]icosasilane [2(C_36_H_30_NP_2_^+^),H_12_Cl_8_Si_20_,C_7_H_7_O_3_S^−^,Cl^−^] [15]. The CSD reference codes are depicted in uppercase letters. Selected bond distances and bond angles associated with the (H−)Si···Cl and/or (X−)Si···X (X = Cl Br, I) contacts are in Å and degree, respectively. Atom labeling is shown for selected atoms.

Figure 1a–f, for example, provides experimental evidence of some molecule–anion systems in the crystalline phase. In them, the halide anion (Cl^−^, Br^−^, or I^−^) attracts the electrophiles on bonded Si atoms in the neutral molecules. Neither anion is located precisely at the centroid of the neutral molecule(s). When the anion (Cl^−^) is entrapped inside the Si_20_ cage of a fully or partially chlorinated icosasilane molecular entity (Figure 1e,f), its position is also off-center so that it maximizes its attraction with all the 20 Si atoms of the Si_20_ cage to stabilize the tetrel-centered (Cl−)Si···Cl and/or (H−)Si···Cl close contacts.

The fundamental phenomena that drive isolated neutral molecules to self-assemble with anions play a significant role in the processes of anion recognition and anion transport, among others [16,17,18]. One such phenomenon is the so-called intermolecular interactions, which are inherently noncovalent [18,19,20].

This study has theoretically examined 25 molecule–anion systems, including their intermolecular geometries, energies, and topological charge-density properties. The molecular entities were the heaviest members, TtI_4_, of the tetrel tetrahalide family (TtX_4_), where Tt stands for the elements in Group 14 of the periodic table and X represents the halide derivative (Tt = C, Si, Ge, Sn, Pb; X = F, Cl, Br, I, At). The anions considered were the halide derivative, X^−^. It is worth mentioning that the theoretical chemistry of 1:1 complexes formed of lighter members of the TtX_4_ (Tt = Si, Ge, Sn) family with the first three halide anions was recently reported [21,22,23,24]. However, the molecule–anion systems considered in this study have never been explored, probably because they were computationally intensive and required a large basis set due to the diffuse character of the heavy atoms involved.

The main purpose of this study is to theoretically clarify the following questions. (i) How strong is the electrophilic region on the electrostatic surface of the Tt atom in molecular TtI_4_? (ii) Can the electrophiles on Tt be active enough to recognize the halide anions when in close proximity? (iii) If so, what would be the strength of the interaction between them? (iv) Should we expect a dependence between descriptors of intermolecular interactions, such as the Tt···X intermolecular distance and the interaction energy for the 25 molecule–anion systems considered? (v) Should the resulting intermolecular interactions between molecular entities responsible for the equilibrium geometries of the [I_4_Tt···X^−^] complexes be called ordinary tetrel bonds [23,25,26,27] or coordinative tetrel bonds [18,28]? A tetrel bond occurs in chemical systems when there is evidence of a net attractive interaction between an electrophilic region associated with a covalently or coordinately bound tetrel atom in a molecular entity and a nucleophilic region in another or the same molecular entity [29]. The chemical origin of a tetrel bond can be intermolecular or intramolecular and is formed by the elements of Group 14 in proximity to a nucleophile.

A number of computational approaches were employed to shed light on the set of questions posed above. The Molecular Electrostatic Surface Potential (MESP) [30,31,32,33] analysis was performed to determine the electrophilic and nucleophilic characters [34,35,36,37,38,39] of specific regions on the surface of each molecular entity, TtI_4_. The Quantum Theory of Atoms in Molecules (QTAIM) [40,41,42,43] and Independent Gradient Model (IGM) [44,45]-based charge density analyses were performed to characterize the shared- and closed-shell characters of intra- and inter-molecular interactions [35,46] responsible for [I_4_Tt···X^−^]. Density functional theory (ωB97X-D [47]) and ab initio calculations (MP2 and CCSD(T)) were performed to obtain geometries, interaction energies, and electronic properties; MP2 and CCSD(T) refer to the second-order Møller–Plesset theory [48,49,50] and Coupled Cluster theory with Singles, Doubles, and Triples excitations [51,52], respectively.

## 2. Computational Methods

The Gaussian 16 [53] calculator was used for the geometric relaxation of the 25 molecule–anion systems; the MP2, CCSD, and ωB97X-D approaches were employed. Two different basis sets were used, including def2-TZVPPD and def2-QZVPPD, obtained from the Basis Set Exchange library [54,55]. It is worth mentioning that we initially planned to use the former pseudopotential, together with the MP2 and ωB97X-D methods, in our calculations, and the MP2 method was chosen based on the results of many previous studies [18,22,56,57]. However, a large discrepancy between the trend in the MP2 and ωB97X-D interaction energies was found for some molecule–anion systems; this was due to the different nature of the tetrel bonding environment and unusually large basis set superposition error (BSSE) encountered with the post Hartree–Fock method. Therefore, we used a relatively large pseudopotential of quadruple zeta valence quality (def2-QZVPPD) to reexamine the MP2 geometries and energies of [I_4_Tt···X^−^]. Computationally expensive CCSD and CCSD(T) methods, in conjunction with the def2-TZVPPD basis set, were also employed to demonstrate the accuracy of MP2 and ωB97X-D geometries and energies of [I_4_Tt···X^−^]. Standard non-relativistic calculations were performed without considering the effect of spin-orbit coupling for heavy atoms such as Pb, following a previous study [17]. Default cutoff criteria for force and displacement for convergence of geometry and frequency calculations were considered. The eigenvalues associated with the normal mode vibrational frequencies of the isolated and complex systems were all positive; thus, the monomer and complex geometries reported are local minima.

To discuss the electrophilicity of the tetrel atom in TtI_4_, the MESP analysis was performed on each of the five isolated monomer geometries. The MESP calculation has utilized wavefunctions generated at the [ωB97X-D/def2-QZVPPD] level. An isoelectron density envelope of 0.001 a.u. was used on which to compute the potential, even though the use of higher isoelectron density envelopes was suggested in other studies for chemical systems containing low-polarizable atomic basins [37,58,59]. We conducted this calculation to obtain the sign and magnitude of local most maxima and minima of potential (*V_S,max_* and *V_S,min_*, respectively) [37,46,60,61,62] on the electrostatic surfaces of molecular TtI_4_. Gaussian 16 [53], Multiwfn [63], and VMD [64] software were used.

Based on the basic concept of MESP [37,58,65,66], if the sign of either *V_S,max_* or *V_S,min_* on a specific region of the molecular surface is positive (i.e., *V_S,max_* > 0 or *V_S,min_* > 0), then the region can be characterized to be electrophilic; if the sign of either *V_S,max_* or *V_S,min_* on a specific region of the molecular surface is negative (i.e., *V_S,max_* < 0 or *V_S,min_* < 0), the region is characterized to be nucleophilic. It is often (but not always!) observed that the sign of *V_S,max_* is positive on the surface of an atom Tt opposite of the R–Tt covalent or coordinating bond, where R is the remaining part of the molecular entity. It occurs when R has a relatively stronger electron-withdrawing capacity than Tt, thus leaving a region of electron-density deficiency on Tt on the opposite side of the R–Tt covalent or coordination bond. This electron-density-deficient region on Tt along the outer extension of the axial direction is referred to as a “σ-hole” [37,38,58,65,67,68]. It should be kept in mind that a σ-hole can be either positive or negative depending on whether *V_S,max_* is positive or negative. The coulombic attraction of an electrophilic σ-hole on the bonded Tt atom in R–Tt and a nucleophile on the same or a different molecular entity is referred to as a σ-hole-centered tetrel bond, or simply σ-hole a interaction [4,23,26,29].

The uncorrected and BSSE corrected interaction energies (*E_int_* and *E_int_(BSSE)*, respectively) of each molecule–anion system were determined using Equations (1) and (2). In Equation (1), *E_T_(complex)*, *E_T_(iso_*1*_)*, and *E_T_(iso_*2*_)* represent the total electronic energies of the molecule–anion complex, isolated molecule, and isolated anion, respectively; in Equation (2), *E(BSSE)* is the error in the total electronic energy due to basis set superposition, obtained using the counterpoise procedure of Boys and Bernardi [69]. The geometry of the isolated molecule in the fully relaxed geometry of the molecule–anion complex was used to obtain *E_T_(iso_*1*_)*.

(1) $$E_{int}=E_{T}\left(complex\right)-E_{T}\left(iso_{1}\right)-E_{T}\left(iso_{2}\right)\;$$

(2) $$E_{int}\left(BSSE\right)=E_{int}+E\left(BSSE\right)$$

QTAIM [40,41,42,43] calculations were performed for 25 molecule–anion systems using [ωB97X-D/def2-QZVPPD] geometries. Five bond descriptors were investigated, including the charge density ρ_b_, the Laplacian of charge density (∇^2^ρ_b_), the gradient kinetic energy density (G_b_), the potential energy density (V_b_), and the total energy density H_b_ (H_b_ = V_b_ + G_b_) at bond critical points. The AIMAll code was used [70].

IGM [44,45]-based calculations were performed at the same theoretical level as QTAIM, and its implications have been actively discussed in various research papers [35,37,46]. The method was originally developed to use promolecular densities to explore the non-covalent chemistry of inter- and intra-molecular interactions in chemical and biological systems [44]. However, using actual densities [45] calculated at the [ωB97X-D/def2-QZVPPD] level, we show that the IGM-δg^inter^-based isosurfaces between interacting atomic basins in [I_4_Tt···X^−^] are consistent with the topological charge-density-based features emanated using QTAIM. Both Multiwfn [63,71] and VMD [64] codes were used.

The delocalization index, δ, is a two-electron property, which is a measure of the number of electron pairs that are being shared between quantum atoms Ω_A_ and Ω_B_ [72,73]. It has also been interpreted as a measure of bond order [74], a property which is formally defined as half the difference between the number of bonding and anti-bonding electrons [75]. We calculated δ within the framework of QTAIM to provide insight into the nature of tetrel bonds in [I_4_Tt···X^−^]. The AIMAll code was used [70].

## 3. Results

### 3.1. The Monomer Properties

Crystals of TtI_4_ (Tt = C, Si, Ge, Sn) have been known since the last century, yet there is no crystallographic evidence of molecular PbI_4_. The crystal structure of the former four species can be retrieved from the Inorganic Crystal Structure Database (ICSD) [76,77,78,79,80]. Table 1 lists the experimental bond distances *r*(Tt–I) and bond angles ∠I–Tt–I of molecular TtI_4_, which are compared with those calculated with [MP2/def2-QZVPPD] and [ωB97X-D/def2-QZVPPD]. The best agreement between experiment and theory is observed with [ωB97X-D/def2-QZVPPD], and MP2 shows a tendency to underestimate *r*(Tt–I). A very similar trend was obtained with these methods in conjunction with the def2-QZVPPD basis set.

**Figure 2:**
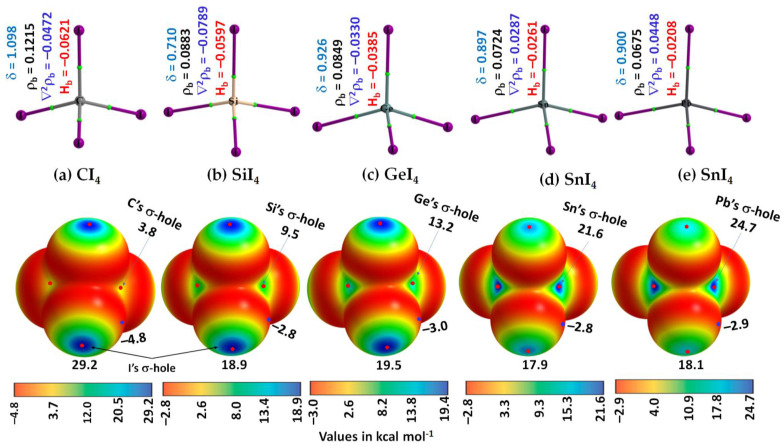
(**Top**) [ωB97X-D/def2-QZVPPD] level QTAIM-based molecular graphs of isolated TtI_4_ (Tt = C, Si, Ge, Sn, and Pb), showing bond paths (solid lines in atom color) and bond critical points (tiny spheres in green) between bonded atomic basins (large spheres). The charge density(ρ_b_), the Laplacian of the charge density (∇^2^ρ_b_), the total energy density (H_b_), and the delocalization index (δ) values are shown in black, blue, red, and faint-blue fonts (in a.u.), respectively. (**Bottom**) The 0.001 a.u. (electrons bohr^−3^) isoelectron density mapped potential on the electrostatic surfaces of the corresponding monomers, including (**a**) CI_4_, (**b**) SiI_4_, (**c**) GeI_4_, (**d**) SnI_4_, and (**e**) PbI_4_. The strength of Tt’s and I’s σ-holes is shown in each case; filled tiny blue and red circles represent *V_S,min_* and *V_S,max_*, respectively; *V_S,min_* and *V_S,max_* values are in kcal mol^−1^.

Figure 2a–e (Top) shows the molecular graph of isolated TtI_4_. From these, it may be seen that the atomic basins are connected to each other in each isolated monomer through bond paths (solid lines between atomic basins in atom color) that pass-through bond critical points (tiny green spheres), thus recovering the expected tetrahedral T_d_ shape of TtI_4_.

The charge density ρ_b_ is larger at the C–I bcps in CI_4_ than at the Pb–I bcps in PbI_4_. It follows the trend across the series: ρ_b_ (C–I) > ρ_b_ (Si–I) > ρ_b_ (Ge–I) > ρ_b_ (Sn–I) > ρ_b_ (Pb–I). The trend signifies that the charge concentration is predominant at the C–I bcps in CI_4_ relative to that at the Pb–I bcps in PbI_4_.

From the sign and magnitude of H_b_, Figure 2a–e (Top), it may be seen that H_b_ is stabilizing (H_b_ < 0) at the Tt–I bcps, which is due to the potential energy density V_b_ that dominates over the gradient kinetic energy density G_b_ at the bcp. H_b_ is increasingly more positive at the Tt–I bcps across the series from CI_4_ through SiI_4_ to GeI_4_ to SnI_4_ to PbI_4_. This is consistent with the character of Tt–I bonds in TtI_4_, in which it progressively becomes less covalent than ionic, passing from CI_4_ through SiI_4_ to PbI_4_. That is, the covalency of the Tt–I bond follows this order: C–I > Si–I > Ge–I > Sn–I > Pb–I. Furthermore, the sign of ∇^2^ρ_b_ at Tt–I bcps is also negative for all monomers except for the Tt–I (Tt = Sn, Pb) bcps, giving an indication that the Tt–I bonds in CI_4_, SiI_4_, and GeI_4_ are relatively more covalent than those in SnI_4_ and PbI_4_. Typically, ∇^2^ρ_b_ < 0 and H_b_ < 0 represent covalent (shared-type) interactions; ∇^2^ρ_b_ > 0 and H_b_ > 0 represent ionic (closed-shell) interactions; and ∇^2^ρ_b_ > 0 and H_b_ < 0 represents mixed (ionic and covalent) interactions [81,82,83,84,85,86].

The delocalization indices, δ, for atom–atom pairs involving Tt and I in TtI_4_ ranged from 0.710 to 1.098, suggesting that they are bound to each other by a σ-type covalent (or coordinate) bond.

**Figure 3:**
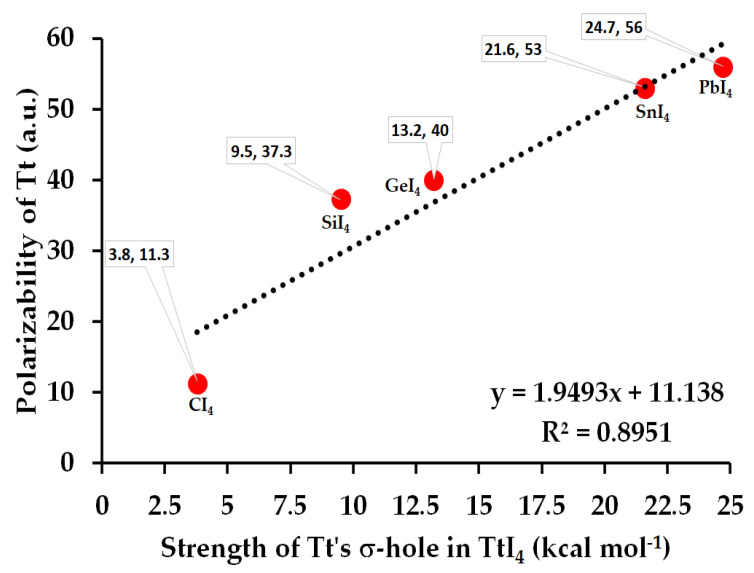
The dependence of polarizability of Tt derivative on the strength of the σ-hole on Tt in TtI_4_, computed using [ωB97X-D/def2-QZVPPD]. The (polarizability, σ-hole) data for each molecule are indicated. The square of the regression coefficient R^2^ is shown, together with the linear equation that connects polarizability with the strength of the σ-hole.

From the MESP graph, Figure 2a–e (Bottom), we observed that there are four σ-holes on each tetrel atom in TtI_4_; they appear along the outer extensions of the four I–Tt covalent/coordinate bonds. They are equivalent for a given tetrel derivative in TtI_4_ (two shown in each case). The strength of the σ-hole is quantified by the local maximum of potential, *V_S,max_*. It varies from 3.8 kcal mol^−1^ (for CI_4_) to 24.7 kcal mol^−1^ (for PbI_4_), revealing that the σ-hole on Tt is electrophilic. The trend in the strength of the σ-hole on Tt in TtI_4_ is in line with the polarizability of the tetrel derivative that increases in the series in this order: C (11.3 a.u.) < Si (37.3 a.u.) < Ge (40.0 a.u.) < Sn (53.0 a.u.) < Pb (56.0 a.u.) [87]. Figure 3 shows the desired relationship between them.

The strength of the σ-hole on each I atom in TtI_4_ along the outer extension of the Tt–I covalent/coordinate bonds is also appreciable. No systematic trend in the strength of the σ-hole on each I atom is observed when passing from CI_4_ through SiI_4_ to GeI_4_ to SnI_4_ to PbI_4_. Because the lateral portions of the covalently bonded I atoms in TtI_4_ are equipped with negative potentials (*V_S,min_* values between −2.8 and −4.8 kcal mol^−1^), each I atom also has a capacity to host as a Lewis base for the attack of an electrophile. These results suggest that TtI_4_ has the ability to function as a donor and acceptor of both tetrel and halogen bonds.

### 3.2. The Complex Properties

**Figure 4:**
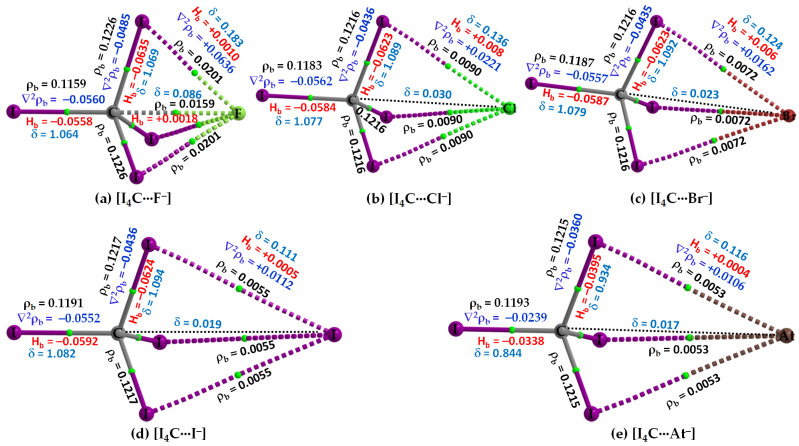
(**a**–**e**) [ωB97X-D/def2-QZVPPD] level QTIM-based molecular graphs of [I_4_C···X^−^], showing the bond paths (solid and dotted lines in atom color) and bond critical points (tiny spheres in green) between bonded atomic basins. Large spheres represent the atomic basins. The dotted black line in (**b**–**e**) is artificially drawn to represent the presence of tetrel bond between C and X (X = Cl, Br, I, At). The charge density (ρ_b_), the Laplacian of the charge density (∇^2^ρ_b_), the total energy density (H_b_), and the delocalization index (δ) values are shown in black, blue, red, and faint-blue fonts (in a.u.), respectively.

**Figure 5:**
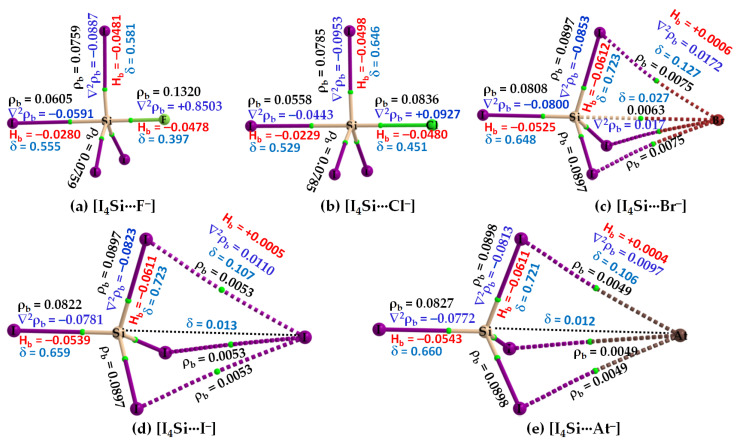
(**a**–**e**) [ωB97X-D/def2-QZVPPD] level QTIM-based molecular graphs of [I_4_Si···X^−^] (X = F, Cl, Br, I, At), showing the bond paths (solid and dotted lines in atom color) and bond critical points (tiny spheres in green) between bonded atomic basins. Large spheres represent the atomic basins, with atoms labeled. The dotted black line in (**d**,**e**) is artificially drawn to represent the presence of tetrel bond between Si and X (X = I, At). The charge density (ρ_b_), the Laplacian of the charge density (∇^2^ρ_b_), the total energy density (H_b_), and the delocalization index (δ) values are shown in black, blue, red, and faint-blue fonts (in a.u.), respectively.

**Figure 6:**
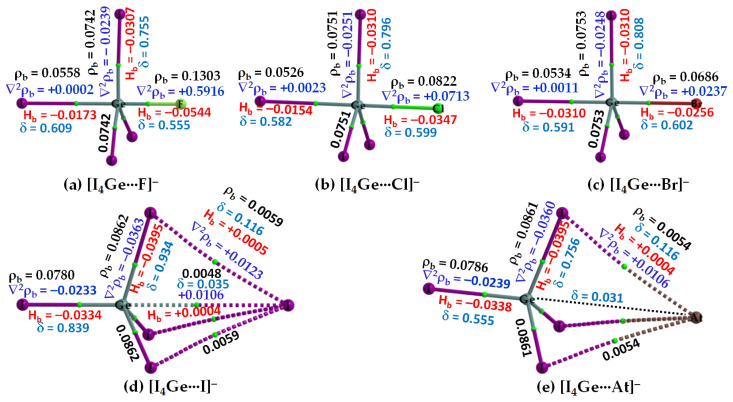
(**a**–**e**) [ωB97X-D/def2-QZVPPD] level QTIM-based molecular graphs of [I_4_Ge···X^−^] (X = F, Cl, Br, I, At), showing the bond paths (solid and dotted lines in atom color) and bond critical points (tiny spheres in green) between bonded atomic basins. Large spheres represent the atomic basins, with atoms labeled. The dotted black line in (**e**) is artificially drawn to represent the presence of tetrel bond between Ge and At. The charge density (ρ_b_), the Laplacian of the charge density (∇^2^ρ_b_), the total energy density (H_b_), and the delocalization index (δ) values are shown in black, blue, red, and faint-blue fonts (in a.u.), respectively.

**Figure 7:**
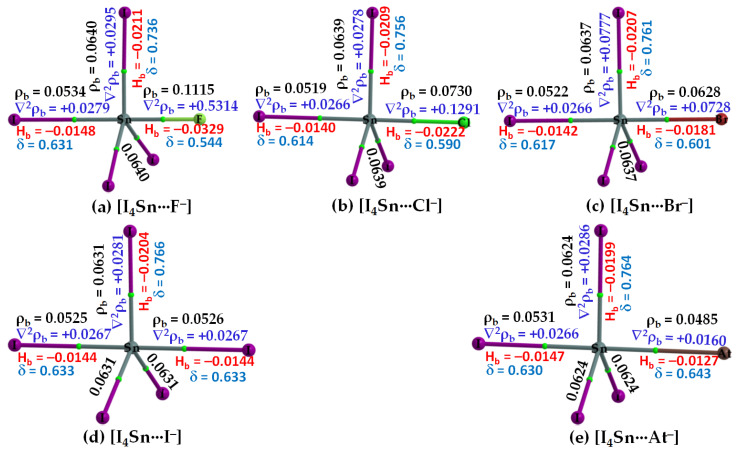
(**a**–**e**) [ωB97X-D/def2-QZVPPD] level QTIM-based molecular graphs of [I_4_Sn···X^−^] (X = F, Cl, Br, I, At), showing the bond paths (solid and dotted lines in atom color) and bond critical points (tiny spheres in green) between bonded atomic basins. Large spheres represent the atomic basins, with atoms labeled. The charge density(ρ_b_), the Laplacian of the charge density (∇^2^ρ_b_), the total energy density (H_b_), and the delocalization index (δ) values are shown in black, blue, red, and faint-blue fonts (in a.u.), respectively.

**Figure 8:**
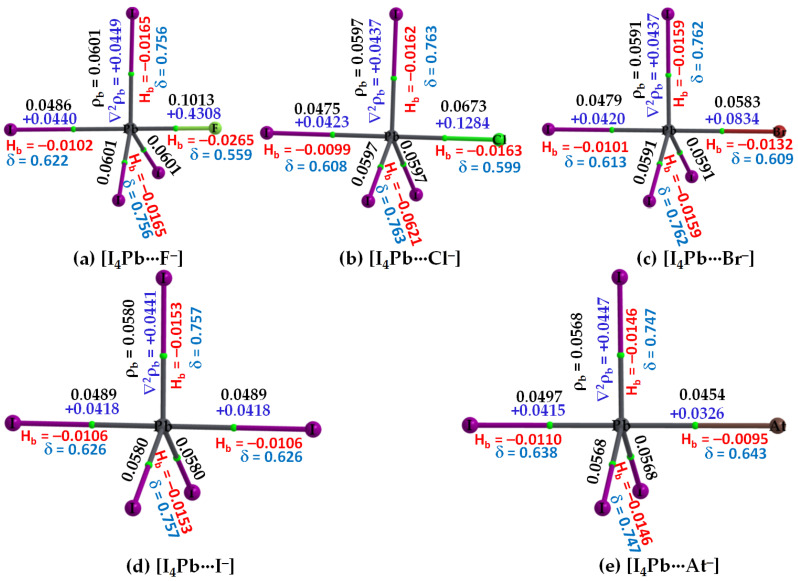
(**a**–**e**) [ωB97X-D/def2-QZVPPD] level QTIM-based molecular graphs of [I_4_Pb···X^−^] (X = F, Cl, Br, I, At), showing the bond paths (solid and dotted lines in atom color) and bond critical points (tiny spheres in green) between bonded atomic basins. Large spheres represent the atomic basins, with atoms labeled. The charge density (ρ_b_), the Laplacian of the charge density (∇^2^ρ_b_), the total energy density (H_b_), and the delocalization index (δ) values are shown in black, blue, red, and faint-blue fonts (in a.u.), respectively.

The five halide anions have linearly approached the Tt atom from the opposite side of the I–Tt covalent bond in TtI_4_, thereby forming [I_4_Tt···X^−^] complexes. They are shown in Figure 4, Figure 5, Figure 6, Figure 7 and Figure 8, in which the Tt···X close contacts were directional (∠I–Tt···X = 180.0°).

#### 3.2.1. The [I_4_C···X^−^] Series

Figure 4 shows the molecular graphs of all the five molecule–anion binary complexes of CI_4_ with the five halide anions. Because the σ-hole on the carbon atom in CI_4_ is the weakest compared to that on the Tt atom in TtI_4_ (Tt = Si, Ge, Sn, Pb) (Figure 2), the strength of the attractive interaction between it and the interacting halide anion(s) is weak-to-strong. For instance, the [CCSD(T)/def2-QZVPPD] level interaction energy is −3.2 and −16.35 kcal mol^−1^ for [I_4_C···F^−^] and [I_4_C···At^−^] (Table 2), respectively. In all cases, the CI_4_ unit in [I_4_C···X^−^], Figure 4, retains its tetrahedral shape similar to that found in its isolated counterpart (Figure 2a, Top).

The C···X intermolecular distance in [I_4_C···X^−^] increases as the halogen derivative becomes more polarizable; it is smallest in [I_4_C···F^−^], with *r*(Tt···F) = 2.665 Å with [CCSD/def2-TZVPPD]. By contrast, the I–C–I angle in complexed [I_4_C···F^−^] either increases or decreases compared to that of its uncomplexed counterpart (∠I–C–I = 109.47°). For instance, the I–C bond linearly attached to the anion forms smaller ∠I–C–I with the three nearest-neighbor I atoms, whereas the remaining I–C bonds that are not directly involved in the formation of tetrel bond are associated with larger ∠I–C–I. These two angles types are 107.5 (111.3°), 108.1 (110.8°), 108.4 (110.5), 108.8 (110.1°), and 108.8 (110.1°) in [I_4_C···F^−^], [I_4_C···Cl^−^], [I_4_C···Br^−^], [I_4_C···I^−^], and [I_4_C···At^−^], respectively.

The bond path and bond critical point features of QTAIM appear between the C and F atomic basins indicate the presence of a C···F tetrel bond in [I_4_C···F^−^], Figure 4a. The appearance of such bond path topologies between F and I atomic basins also indicates the presence of three equivalent I···F close contacts in [I_4_C···F^−^]. The latter ones mimic the I···X (X = Cl, Br, I, At) links in the other four members of the same family (Figure 4b–e). However, QTAIM did not reveal the presence of any Tt···X tetrel bonded contacts in [I_4_C···X^−^] when X = Cl, Br, I, and At. Although this kind of σ-hole interaction is expected based on inferences from the results of the MESP model (Figure 2, Bottom), their absence in the QTAIM molecular graph may be due to the stringent nature of boundaries between bonded atom basins as determined by the arbitrary nature of the space partitioning approach. Similar observations have been reported in several previous instances that do [88] or do not [89,90,91] involve tetrel bonding.

The authenticity of the I···X interactions in [I_4_C···X^−^] is confirmed by the I and X intermolecular distances that are close to the sum of their respective van der Waals radii (vdW), a feature that has been recommended for identifying hydrogen bonds [92], halogen bonds [93], chalcogen bond [94], pnictogen bond [35,39,62,95], tetrel bonds [27], and any other noncovalent interactions [96]. In any case, the I···F, I···Cl, I···Br, I···I and I···At intermolecular distances in [I_4_C···F^−^], [I_4_C···Cl^−^], [I_4_C···Br^−^], [I_4_C···I^−^], and [I_4_C···At^−^] are ca. 2.871, 3.626, 3.892, 4.274, and 4.322 Å, respectively. The former three are less than their respective sum of vdW radii of 3.50 (I + F), 3.86 (I + Cl), and 3.90 Å (I + Br), whereas the latter two are slightly greater than the sum of their respective sum of vdW radii of 4.08 (I + I) and 4.04 Å (I + At). (vdW radii of atoms were taken from ref. [97], except for At, which was taken from ref [98]). Since the vdW radii of atoms are accurate within an uncertainly of ±0.2 Å [35,36,37,39,46,62,97,99], the possibility of I···X (X = I, At) close contacts in [I_4_C···I^−^] and [I_4_C···At^−^] that were revealed by QTAIM are not misleading, Analogous halogen···halogen interactions in some chemical systems are known [100,101], which have been interpreted as unusually strong vdW type [101].

Both ωB97X-D and MP2 have predicted an analogous bonding scenario in [I_4_C···X^−^], as CCSD. However, the increase in the size of the basis set from def2-TZVPPD to def2-QZVPPD has resulted in a slight increase in the Tt···X intermolecular distance with ωB97X-D and MP2. Whatever is the size of the basis set, the intermolecular distances predicted using MP2 are underestimated relative to ωB97X-D and CCSD. Furthermore, [MP2/def2-TZVPPD] has predicted the C···I and C···At bond distances to be 4.091 and 4.083 Å for [I_4_C···I^−^] and [I_4_C···At^−^], respectively; they were 3.990 and 4.009 Å with def2-QZVPPD, respectively. This means that MP2 does not correctly predict the trend in C···I and C···At bonding distances, as predicted by ωB97X-D and CCSD (cf. Table 2).

The three I–C bonds in I_4_C, which are not directly involved with the halide anions in [I_4_C···X^−^] to form the C···X tetrel bond, are equivalent. Accordingly, each of the three properties, such as ρ_b_, ∇^2^ρ_b_ (∇^2^ρ_b_ < 0) and H_b_ (H_b_ < 0) at the bcps of the three equivalent bonds, were equivalent (one shown for each complex in Figure 4). The I–C bond in I_4_C, which is responsible for the formability of the C···X tetrel bond, is largely affected in [I_4_C···F^−^] compared to that in the other four members of the series and the charge density at the I–C bcp is decreased as a result of elongation of the bond. However, for all cases, all the four I–C bonds in tetrahedral I_4_C is covalent since H_b_ < 0 and ∇^2^ρ_b_ < 0 at the I–C bcps. δ for these bonds are ranging between 0.8 and 1.1, giving evidence of their single-bond character. By contrast, H_b_ > 0 and ∇^2^ρ_b_ > 0 for the C···F bcp in [I_4_C···F^−^], and I···X bcps in [I_4_C···X^−^], and the charge density is very small at corresponding bcps. Moreover, δ for the atom–atom pairs responsible for the C···X tetrel bond decrease systematically in the series in this order: [I_4_C···F^−^] (δ = 0.086) > [I_4_C···Cl^−^] (δ = 0.030) > [I_4_C···Br^−^] (δ = 0.023) > [I_4_C···I^−^] (δ = 0.019) > [I_4_C···At^−^] (δ = 0.017), in consistent with the trend found for interaction energy (Table 2).

#### 3.2.2. The [I_4_Tt···X^−^] (Tt = Si, Ge) Series

The nature of the intermolecular bonding environment found in [I_4_C···X^−^], Figure 4, is not the same for all the five members of the [I_4_Si···X^−^] or [I_4_Ge···X^−^] series (cf. Figure 5 and Figure 6, respectively). Because the electrostatic surfaces of Si and Ge in SiI_4_ and GeI_4_, respectively, were relatively more electrophilic than that on C in CI_4_, they showed reasonably strong selectivity for the anions. This was specifically true when X pointed to F, and Cl (Figure 5a,b) but not when X pointed to Br, I, and At in [I_4_Si···X^−^] (Figure 5c–e). Similarly, the Ge atom in GeI_4_ appreciably recognizes the halide anions when X is F, Cl, or Br (Figure 6a–c), but not when X is I, or At in [I_4_Ge···X^−^] (Figure 6d,e). This means that the strength of Tt···X bonding is moderate when the latter two heavy halide anions are involved, in which the degree of tetrahedral deformation of the I_4_Si/I_4_Ge unit in [I_4_Si···X^−^]/[I_4_Ge···X^−^] is small. When the I_4_Si/I_4_Ge isolated monomers strongly recognize the halide anions, the degree of deformation of I_4_Si/I_4_Ge is overwhelmingly large, and hence the tetrahedral shape of SiI_4_/GeI_4_ in [I_4_Si···X^−^]/[I_4_Ge···X^−^] (X = F, Cl, or Br) is completely lost.

While the bonding features noted above were obtained from [ωB97X-D/def2-QZVPPD], the [CCSD/def2-QZVPPD] method has predicted an exception for [I_4_Si···Br^−^], in which, the responsible interacting units were involved in the formation of a dative tetrel bond; it is in a manner similar to that found for [I_4_Si···X^−^] (X = F, Cl). On the other hand, MP2 has recognized the attraction between I_4_Si and X^−^ in the first four members of the [I_4_Si···X^−^] series to be unusually strong and that in [I_4_Si···At^−^] to be moderate. The former result with MP2 is applicable to the [I_4_Ge···X^−^] series as well. This means that the Tt···X close contacts in the above-mentioned molecule–anion systems are not ordinary tetrel bonds; they are dative tetrel bonds.

Our QTAIM analysis, Figure 6a–c, revealed that ρ_b_ is appreciable at the Ge···X bcps in [I_4_Ge···X^−^] when X points to F, Cl, and Br. For [I_4_Ge···I^−^], the ρ_b_ is small at Ge···I bcp (ρ_b_ = 0.0048 a.u.), and the interaction between the monomers is also reinforced by I···I interactions (Figure 6d). The ρ_b_ values at the Ge···X (X = F, Cl, Br) bcps in [I_4_Ge···X^−^] are not only typical for coordinate bonds but larger than that can be expected for ordinary non-covalent interactions such as hydrogen bonds, and halogen bonds, among others (ρ_b_ < 0.05 a.u.). They may be comparable with the ρ_b_ values of the Tt–I coordinate bonds in isolated and complexed TtI_4_. A similar conclusion might be arrived at for Si···X bcps in [I_4_Si···X^−^] (X = F, Cl), Figure 5a,b.

From the sign and magnitude of ∇^2^ρ_b_ (∇^2^ρ_b_ > 0) and H_b_ (H_b_ < 0), Figure 6a–c, it is realized that the Ge···X (X = F, Cl, Br) tetrel bonds possess mixed ionic and covalent character. This view is also transferable to the Si···X (X = F, Cl, Br) tetrel bonds in [I_4_Si···X^−^] provided [CCSD/def2-TZVPPD] results are considered. The large δ values corresponding to atom–atom pairs responsible for the Si···X and Ge···X (X = F, Cl, Br) close contacts provide further evidence that there are no π-type interactions involved; they are purely σ-type coordinate dative bonds. By contrast, the δ values are very small for atom–atom pairs causing the Si···X and Si···X close contacts in [I_4_Ge···X^−^] and [I_4_Ge···X^−^] (X = I, At), respectively, indicative of closed-shell interactions. The three equivalent I···X halogen···halogen close contacts in each of [I_4_Ge···X^−^] and [I_4_Ge···X^−^] (X = I, At) are described by small δ values, and positive ∇^2^ρ_b_ and H_b_. Similarly, the Si···X and Ge···X (X = I, At) tetrel bonds are described by small δ values, as expected.

#### 3.2.3. The [I_4_Tt···X^−^] (Tt = Sn, Pb) Series

The σ-holes on Sn and Pb in SnI_4_ and PbI_4_, respectively, are stronger than those of TtI_4_ (Tt = C, Si, Ge). Therefore, their acidic strengths are adequate enough to recognize the five halide anions when in close proximity. This may be rationalized from QTAIM’s molecular graphs of resulting configurations, [I_4_Sn···X^−^] and [I_4_Pb···X^−^] (X = F, Cl, Br, I, At), illustrated in Figure 7 and Figure 8, respectively. As can be seen, the formation of intermolecular interaction in each of them has caused profound damage to the tetrahedral framework of isolated SnI_4_ and PbI_4_. This means that the TtI_4_ molecule is structurally fully deformed in the presence of each of the five halide anions. There is no secondary intermolecular interaction that can play a role in the geometrical stability of the resulting complex anions, as found for other series (see above). In all cases, the tetrel center adopts a trigonal bipyramidal geometry (a molecular structure with one atom at the center and five more atoms at the corners of the trigonal bipyramid). Clearly, the resulting complex anions each is nothing but a coordination compound, and the Tt···X close-contact is formally a Tt–X dative tetrel bond. In such cases, charge transfer from the anion to the σ*(I–Tt) anti-bonding orbital is expected, and the S_N_2 mechanism is likely to play a role in driving the dative bond formation between the interacting species [23].

Our calculation suggests that the extent of charge transfer is the largest for the [I_4_Pb···X^−^] series and the smallest for the [I_4_C···X^−^] series. In particular, the [ωB97X-D/def2-QZVPPD] level QTAIM charge transfer from X^−^ to PbI_4_ is 0.263, 0.371, 0.424, 0.515 and 0.588 *e* for [I_4_Pb···F^−^], [I_4_Pb···Cl^−^], [I_4_Pb···Br^−^], [I_4_Pb···I^−^], and [I_4_Pb···At^−^], respectively. The corresponding charge transfer values were 0.229, 0.334, 0.382, 0.460, and 0.519 *e* for [I_4_Sn···F^−^], [I_4_Sn···Cl^−^], [I_4_Sn···Br^−^], [I_4_Sn···I^−^], and [I_4_Sn···At^−^], respectively. These results imply that the nature of charge-transfer in the Sn- and Pb-based anions is virtually similar and that the charge-transfer preference is consistent with the interaction energy preference across a given series, indicating that the charge-transfer phenomenon is likely to be one of the most prominent contributors to the interaction. 

The above nature of charge transfer is moderately large, for example, relative to that of 0.115, 0.088, 0.082, 0.077, and 0.076 *e* calculated for [I_4_C···F^−^], [I_4_C···Cl^−^], [I_4_C···Br^−^], [I_4_C···I^−^], and [I_4_C···At^−^], respectively. These results may lead to a conclusion that the formation of stronger complexes accompanies an appreciable amount transfer of charge between the interacting monomers, and is not very surprising [102].

From the molecular graphs of QTAIM in Figure 7 and Figure 8, it seems that the charge density at Tt···X bcps between I_4_Tt and X^−^ in [I_4_Tt···X^−^] (Tt = Sn, Pb; X = F, Cl, Br, I, At) is non-negligible; it may be comparable to that of the Tt–I bcps of complexed I_4_Tt. The Tt–I and Tt···X bcps are both characterized by ∇^2^ρ_b_ > 0 and H_b_ < 0, indicating the presence of a mixed covalent and ionic character. Since H_b_ becomes increasingly more positive at the Tt···X bcp passing from [I_4_Tt···F^−^] through [I_4_Tt···Cl^−^] to [I_4_Tt···Cl^−^] to [I_4_Tt···Br^−^] to [I_4_Tt···I^−^] to [I_4_Tt···At^−^], it is clear that these interactions are less covalent in the same order. The ∇^2^ρ_b_ values are decreasing in the series from [I_4_Tt···F^−^] through [I_4_Tt···Cl^−^] to [I_4_Tt···At^−^], indicating that the Tt···F tetrel bond is more ionic than the Tt···At tetrel bond. In the case of Tt = Si and Ge, the Tt···X (X = F, Cl, Br) bcps show ∇^2^ρ_b_ > 0 and H_b_ < 0 (Figure 5 and Figure 6). The four Si–I bonds in I_4_Si of [I_4_Si···X^−^] are potentially covalent since ∇^2^ρ_b_ < 0 and H_b_ < 0 at the bcps of these bonds, as like as the three Ge–I bonds in I_4_Ge of [I_4_Ge···X^−^] that are orthogonal to the Tt···X (X = F, Cl, Br) bond in the respective system. The characteristics of Si–I bonds in I_4_Si resemble the C–I bonds in I_4_C.

The δ of the atom–atom pairs for Tt–I and Tt···X bonds in [I_4_Tt···X^−^] (Tt = Sn, Pb) is considerably larger than what were calculated for ordinary tetrel bonds (see Figure 6a–c for the former and Figure 6d–e for the latter bonds, for example). It is considerably smaller than those in isolated I_4_Tt (Figure 2, Top), thus consistent with the weakening of the Tt–I bond in I_4_Tt (Tt = Si, Ge, Sn, Pb) upon its attractive engagement with the halide anions. For comparison, we note that the δ values for Tt–I and Tt···X coordinate and dative tetrel bonds in [I_4_Tt···X^−^] are smaller than, and comparable to, those reported for metal–C(O) coordinate bonds (δ values ranged from 0.279 to 1.195); however, those of Tt···X ordinary tetrel bonds are comparable with what were reported for metal···metal (metal···H or H···H) interactions (δ values between 0.005 and 0.166) in [M_2_(CO)_10_] and [M_3_(μ-H)_3_(CO)_12_] (M = Mn, Tc, Re) complexes [103]. 

#### 3.2.4. IGM-δg^inter^ Analysis

The formation of [I_4_Tt···X]^−^ has caused the weakening of all the four Tt–I bonds in TtI_4_, compared to that found in the uncomplexed TtI_4_. The weakening was evidence of the elongation of the Tt–I bonds in [I_4_Tt···X]^−^. Concomitant with the elongation was the decrease in the charge density at the Tt–I bcps, which may be inferred comparing the ρ_b_ values at the Tt–I bcps shown in Figure 2 (Top) for isolated TtI_4_ and in Figure 4, Figure 5, Figure 6, Figure 7 and Figure 8 for complexed TtI_4_. Th existence of I···X and Tt···X in some complexes of [I_4_Tt···X]^−^ (Tt = C, Si, and Ge) are also genuine, which are confirmed by the results of IGM-δg*^inter^* analysis.

**Figure 9:**
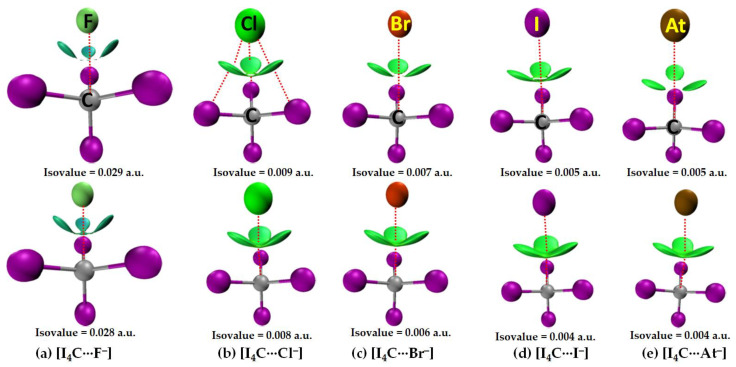
(**a**–**e**) [ωB97X-D/def2-QZVPPD] level IGM-*δg^inter^*-based isosurface (bluish-green or green volumes) plots of [I_4_C···X^−^] (X = F, Cl, Br, I, At), showing possible I···X halogen bonded and C···X tetrel bonded interactions between interacting molecular entities. (Top) Illustration of I···X Type-I halogen-halogen bonded interactions between the interacting units that appear with larger isovalues. (Bottom) Illustration of C···X tetrel bonded interactions between the interacting units that appear at smaller isovalues. Anion derivatives are labeled.

Figure 9 illustrates the results of IGM-*δg^inter^* analysis for [I_4_Tt···X^−^] (Tt = C). The I···X closed contacts in several of these systems appeared at larger IGM-*δg^inter^* isovalues (Top). On the other hand, the Tt···X close contacts showed up at lower IGM-*δg^inter^* isovalues (Bottom). This is not very surprising since smaller isolvalues are typically necessary for the physical appearances of isosurfaces corresponding to weakly bonded interactions. By contrast, the relatively stronger interactions can be traceable with larger isovalues since charge density around the critical bonding region is generally appreciable. The bluish isosurface originated with large IGM-*δg^inter^* isovalues for [I_4_C···F^−^] indicates that the attraction between the interacting units is very prominent (Figure 9, Bottom). When the size of the halogen derivative increases, the attraction between C and X in [I_4_C···X^−^] weakens, and hence, the isosurfaces become increasingly greenish (Figure 9, Bottom). These results are concordant with the nature of the QTAIM-based charge density features at the I···F and C···F bcps (cf. Figure 4). Therefore, the stabilization of [I_4_C···X^−^] is not just due to the formation of the C···X tetrel bonds alone but partly arises from the I···X Type-I halogen···halogen bonded interactions as well.

**Figure 10:**
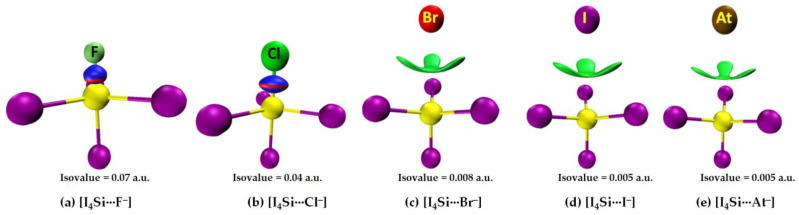
(**a**–**e**) [ωB97X-D/def2-QZVPPD] level IGM-*δg^inter^*-based isosurface (bluish-red or green volumes) plots for [I_4_Si···X^−^] (X = F, Cl, Br, I, At), showing possible I···X halogen-halogen bonded and Si···X tetrel bonded interactions between interacting molecular entities.

The [I_4_Si···X^−^] systems feature very similar IGM-*δg^inter^*-based isosurfaces, Figure 10. That is, a very large isovalue was necessary to reveal sizable isosufaces describing the dative tetrel bond in [I_4_Si···F^−^] and [I_4_Si···Cl^−^], whereas a potentially small isovalue was required to visualize the isosurface domains in [I_4_Si···X^−^] (X = Br, I, At). The view is also transferable to the [I_4_Ge···X^−^] systems (not shown).

**Figure 11:**
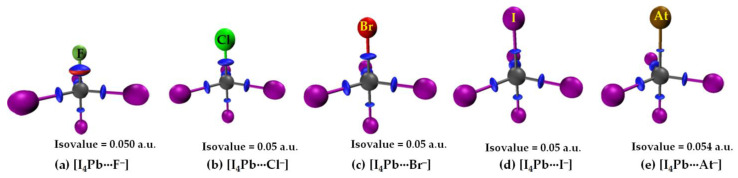
(**a**–**e**) [ωB97X-D/def2-QZVPPD] level IGM-*δg^inter^*-based isosurface plots (bluish-red volumes) of [I_4_Pb···X^−^] (X = F, Cl, Br, I, At), showing possible Pb···X tetrel bonded interactions between interacting molecular entities.

In the case of [I_4_Sn···X^−^] and [I_4_Pb···X^−^] (X = F, Cl, Br, I, At), the IGM-*δg^inter^* isosurfaces were visualizable with an isovalue close to 0.055 a.u. Figure 11 shows this for the [I_4_Pb···X^−^] series. Passing from the left to the right of Figure 11, it can be seen that the thickness and size of the bluish isosurface volume describing the dative tetrel bond between Tt and X are decreasing. This result is also in agreement with QTAIM in that the charge density between these atomic basins decreases from [I_4_Tt···F^−^] through [I_4_Tt···Cl^−^] to [I_4_Tt···Br^−^] to [I_4_Tt···I^−^] to [I_4_Tt···At^−^] (Tt = Sn, Pb).

#### 3.2.5. Interaction Energies

Except for the [I_4_C···X^−^] series and [I_4_Tt···X^−^] (Tt = Si, Sn; X = I, At), the *E_int_ (BSSE)* values for all other molecule–anion systems are much larger than the so-called covalent limit for hydrogen bonds (−40.0 kcal mol^−1^) [104,105,106]. From the values of the interaction energies of 25 molecule–anion complexes, [I_4_Tt···X^−^], it is clear that the complex stability is largely determined by the polarizability of the Tt atom in I_4_Tt and the halogen derivative. These energies calculated in the range from −3.0 to −112.2 kcal mol^−1^ with [CCSD(T)/def2-TZVPPD], Table 2, can be categorized as weak (−3.0 kcal mol^−1^ < *E_int_ (BSSE)* < −5.0 kcal mol^−1^), moderate (−5.0 kcal mol^−1^ < *E_int_ (BSSE)* < −10.0 kcal mol^−1^), strong (−10.0 kcal mol^−1^ < *E_int_ (BSSE)* < −25.0 kcal mol^−1^), very strong (−25.0 kcal mol^−1^ < *E_int_ (BSSE)* ≤ −40.0 kcal mol^−1^), and ultra-strong (*E_int_ (BSSE)* >> −40.0 kcal mol^−1^ (the covalent limit for hydrogen bond). At the highest level of theory applied, [CCSD(T)/def2-TZVPPD], the weakest and strongest of the [I_4_Tt···X^−^] systems are found to be [I_4_C···At^−^] (*E_int_ (BSSE)* = −3.20 kcal mol^−1^) and [I_4_Si···F^−^] (*E_int_ (BSSE)* = −112.15 kcal mol^−1^), respectively.

From Table 2, two major differences are noteworthy. First, ωB97X-D predicts a BSSE-corrected interaction energy of −9.61 and −9.80 kcal mol^−1^ for [I_4_Si···Br^−^] with def2-TZVPPD and def2-QZVPPD, respectively; these are indicative of the fact that the strength of the tetrel bond between Si of I_4_Si and Br^−^ is moderate. As mentioned already above, this is not the case with MP2 since the *E_int_* (BSSE) for the same system, for instance, with def2-QZVPPD, is predicted to be −60.0 kcal mol^−1^; the large *E_int_* (BSSE) implies that the attraction between I_4_Si and Br^−^ causes the formation of Si–Br dative tetrel bond. This result is consistent with [CCSD(T)/def2-TZVPPD], which has predicted an *E_int_* (BSSE) of −53.16 kcal mol^−1^ for the same system. Second, the [ωB97X-D/def2-TZVPPD] level *E_int_* (BSSE) values for the remaining four systems of the [I_4_Si···X]^−^ series are in qualitative and quantitative agreement with [CCSD(T)/def2-TZVPPD]. MP2, however, unusually overestimated the interaction energies for [I_4_Si···I^−^] and [I_4_Si···At^−^]. The discrepancy between the DFT (or CCSD(T)) and MP2 energies is likely due to the latter method’s misleading prediction of the Si···I and Si···At close contacts, thus pushing the interacting atoms in [I_4_Si···I^−^] to be bonded with each other via a dative tetrel bond. These peculiar results indicate that applying the MP2 approach to predict the correct nature of the tetrel bond in molecule–anion complex systems formed by heavier tetrel derivatives in molecular entities should be exercised with caution.

The preference of BSSE-corrected interaction energy, *E_int_(BSSE),* between the five members of each series [I_4_Tt···X]^−^ follows the trend: [I_4_Tt···F^−^] > [I_4_Tt···Cl^−^] > [I_4_Tt···Br^−^] > [I_4_Tt···I^−^] > [I_4_Tt···At^−^] (Table 2). This is the energy preference at the highest level of theory applied, [CCSD(T)/def2-TZVPPD], which shows a tendency for the strength of the interaction to decrease with increasing polarizability of the halogen derivative (F < Cl < Br < I < At). This stability preference could not be reproduced with ωB97X-D when def2-TZVPPD was used since it altered the stability priority between [I_4_Tt···I^−^] and [I_4_Tt···At^−^] when Tt = C and Pb, giving rise to: [I_4_Tt···F^−^] > [I_4_Tt···Cl^−^] > [I_4_Tt···Br^−^] > [I_4_Tt···I^−^] ≤ [I_4_Tt···At^−^]. The same trend was also observed when MP2 was used in conjunction with def2-NZVPPD (N = T, Q). Note that changing the basis set from def2-TZVPPD to def2-QZVPPD somehow restored the [CCSD(T)/def2-TZVPPD] level energy preference at the ωB97X-D level (but not at the MP2 level) when Tt = C, but not when Tt = Pb. One reason for the anomalous change in the preference of energy ordering between [I_4_Tt···I^−^] and [I_4_Tt···At^−^] is that the post-HF MP2 method greatly overestimates the BSSE, as well as the electron-electron correlation energy, relative to the DFT and CCSD(T). On the other hand, the CCSD(T) method has properly accounted for electron-electron correlation energy, which ensured the correct preference of stabilization energies among the five members of any given series, [I_4_Tt···X^−^].

**Figure 12:**
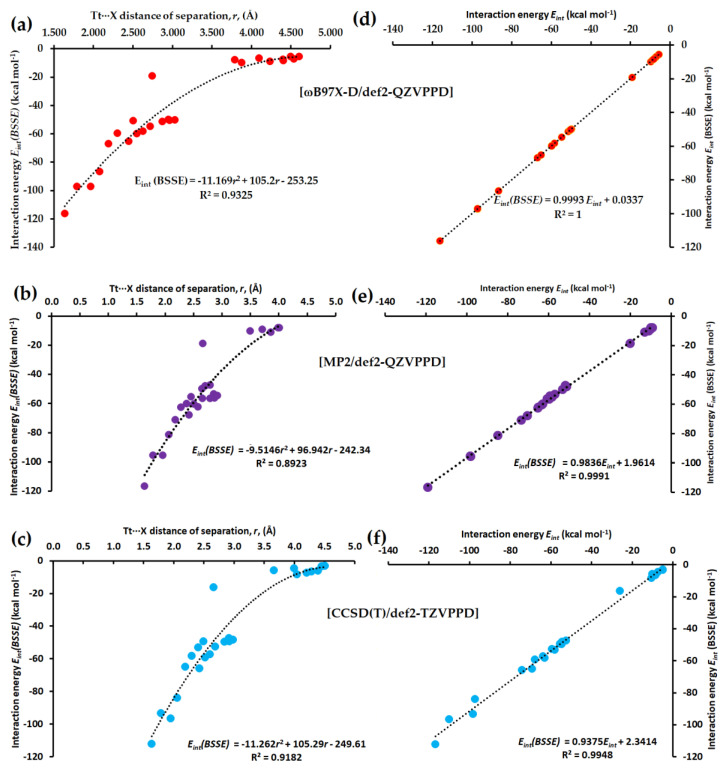
(**a**) The [ωB97X-D/def2-QZVPPD] level quadratic dependence of BSSE corrected interaction energy (*E_int_(BSSE)*) on the distance of separation *r*(Tt···X) for 25 [I_4_Tt···X^−^] (Tt = C, Si, Ge, Sn, Pb; X = F, Cl, Br, I, At) molecule–anion complexes. Shown in (**b**,**c**) are the corresponding dependences obtained using [MP2/def2-QZVPPD] and [CCSD(T)/def2-TZVPPD], respectively. Shown in (**d**–**f**) are the linear dependences between *E_int_(BSSE)* and *E_int_* obtained with [ωB97X-D/def2-QZVPPD], [MP2/def2-QZVPPD] and [CCSD(T)/def2-TZVPPD], respectively.

Figure 12a–c compares the type of dependence of *E_int_(BSSE)* on the distance of separation *r*(Tt···X) for 25 molecule–anion complexes, [I_4_Tt···X^−^], obtained using ωB97X-D, MP2 and CCSD(T). Regardless of the different calculation methods utilized, the dependence was found to be quadratic. The square of the regression coefficient R^2^ was moderately higher (R^2^ = 0.9325) for ωB97X-D compared to CCSD(T) and lower (R^2^ = 0.8923) for MP2.

We note further that the BSSE in energy is minimal with DFT but larger with MP2 and CCSD(T). It is very large with the def2-TZVPPD basis set than with the def2-QZVPPD basis set. For example, for ωB97X-D, MP2, and CCSD(T) with def2-TZVPPD, the BSSE in energy ranged from 0.05 to 9.68 kcal mol^−1^, from 2.60 to 13.58 kcal mol^−1^, and from 1.42 to 13.44 kcal mol^−1^, respectively. However, when using the def2-QZVPPD basis set, the BSSE in energy has decreased sharply, giving rise to values in the range from 0.02 to 0.13 kcal mol^−1^ with ωB97X-D and from 1.01 to 5.01 kcal mol^−1^ with MP2. CCSD(T) with def2-QZVPPD was computationally very expensive; no conclusions could be drawn about the range of BSSE in energy with this method. Figure 12d–f compares the nature of dependence between *E_int_(BSSE)* and *E_int_*, obtained using [ωB97X-D/def2-QZVPPD], [MP2/def2-QZVPPD] and [CCSD(T)/def2-TZVPPD], respectively, showing a perfect linear dependence at the former level than that at the latter two. 

## 4. Discussion and Concluding Remarks

In this study, the series [I_4_Tt···X^−^] (Tt = C, Si, Ge, Sn, Pb; X = F, Cl, Br, I, At) was theoretically investigated to clarify the nature of the selectivity of the I_4_Tt host for five guest (halide) anions. The MP2 geometries and interaction energies for the 25 molecule–anion systems were underestimated and overestimated, respectively, relative to DFT and CCSD methods, and in some cases, the MP2 results were unreliable. The chemical bonding features obtained using DFT were consistent with the computationally expensive CCSD and CCSD(T) results, with an exception for [I_4_Si···Br^−^]. For the latter, the tetrel bonding characteristics predicted by CCSD could not be reproduced by DFT-ωB97X-D. Similarly, the significant overestimation of the interaction energy of [I_4_Si···I^−^] with MP2 was in sharp disagreement with that computed using ωB97X-D and CCSD(T).

The deformation of the tetrahedral skeletal framework of TtX_4_ was shown to be prominent, especially when the electron-withdrawing anions, viz. F^−^ and Cl^−^, and sometimes Br^−^, were used as partner interacting species for TtI_4_ (Tt = Si, Ge, and Sn). When Sn and Pb of TtI_4_ were acted as tetrel bond donors for all the five anions, the original tetrahedral shape of parent TtI_4_ was completely lost, and the tetrel atoms in the resulting complex anion systems preferred adopting a pentagonal bipyramidal geometry. This was attributed to the strong electrophilicity (greater selectivity) of the heavier tetrel derivative, in which appreciable charge transfer occurred from the anion to the tetrel donor moiety that led to dative tetrel bond formation.

In several complexes, tetrel bonding did not occur alone. This was true especially when the molecule hosting the tetrel bond donor was not fully deformed. In this case, the same anion that caused the formation of an ordinary Tt···X tetrel bond was simultaneously engaged with three nearest-neighbor iodine atoms that were responsible for an I_3_ face of the TtI_4_ tetrahedron. This also means that the strength of the Tt···X tetrel bond in these complex systems might be reinforced by the I···X interactions, evidenced by the charge-density-based topological results of QTAIM and IGM-δg*^inter^*. These results enable us to believe that a similar intermolecular bonding scenario might be existing between the interacting monomers responsible for some of the systems of the series [Y_4_Tt···X^−^] (Tt = C, Si, Ge; Y = F, Cl Br) [21]; further computational studies on them are a prerequisite to validate our claim.

The Tt···X separation distance calculated by [CCSD/def2-TZVPPD] was smaller than the vdW radii of the Tt and X atoms for all [I_4_Tt···X^−^] systems, except for [I_4_C···X^−^] (X = Cl, Br, I, At) and [I_4_Si···X^−^] (X = I, At). This was the case with [ωB97X-D/def2-TZVPPD] and [ωB97X-D/def2-QZVPPD], but [I_4_Ge···At^−^] was added to the exclusion list. The result was different from [MP2/def2-QZVPPD] in that it predicted exceptions only for [I_4_C···X^−^] (X = Br, I, At), and is not surprising given it is an MP2’s tendency to underestimate intermolecular distances. Although these latter two computational methods exclude intermolecular interactions in systems that do not follow the stringent “less than the sum of vdW radii rule,” the exclusion was also consistent with the nature of bond path topology revealed using QTAIM. For example, for [I_4_C···X^−^] (X = Cl, Br, I, At), [I_4_Tt···X^−^] (Tt = Si; X = I, At) and [I_4_Tt···X^−^] (Tt = Ge; X = At), no QTAIM-based bond path topology exists between Tt and X at the [ωB97X-D/def2-QZVPPD] level. This means that QTAIM does not recognize the existence of Tt···X tetrel bonding in the host–guest systems when the tetrel bond distance between Tt and X exceeds the sum of the vdW radii of Tt and X, even though this type of limitation of QTAIM has been attributed to the arbitrary nature of the space partitioning scheme. Even so, it should be borne in mind that the Tt and X atoms of the interacting monomers in all complex systems were indeed tetrel bonded to each other, evidenced by the IGM-*δg^inter^*-based isosurfaces.

## Figures and Tables

**Table 1:** Comparison of computed Tt–I bond distances *r* (Å) and I–Tt–I bond angles ∠ (degree) of TtI_4_ (Tt = C, Si, Ge, Sn, and Pb) with those feasible in their corresponding crystals retrieved from the Inorganic Crystal Structure Database (ICSD) ^a^.

Monomer	Property	Expt. ^a^	[MP2/def2-QZVPPD]	[ωB97X-D/def2-QZVPPD]
CI_4_	*r*(C–I)	2.154	2.131	2.254
	∠I–C–I	109.47	109.47	109.47
SiI_4_	*r*(Si–I)	2.434	2.403	2.434
	∠I–Si–I	109.47	109.47	109.47
GeI_4_	*r*(Ge–I)	2.574	2.463	2.518
	∠I–Ge–I	109.47	109.47	109.47
SnI_4_	*r*(Sn–I)	2.650	2.463	2.518
	∠I–Sn–I	109.47	109.47	109.47
PbI_4_	*r*(Pb–I)	---	2.705	2.749
	∠I–Pb–I	---	109.47	109.47

^a^ CI_4_ (ICSD ref: 30789); SiI_4_ (ICSD ref: 22100); GeI_4_ (ICSD ref: 22399); and SnI_4_ (ICSD ref: 18010).

**Table 2:** Comparison of ωB97X-D and MP2 level intermolecular bond distances, uncorrected and BSSE corrected interaction energies of [I_4_Tt···F^−^] (Tt = C, Si, Ge, Sn, Pb; X = F, Cl, Br, I, At) with those calculated with CCSD and CCSD(T) ^a^.

System	[ωB97X-D/def2-TZVPPD]			[ωB97X-D/def2-QZVPPD]			[MP2/def2-QZVPPD]			[CCSD(T)/def2-TZVPPD]		
	*E_int_*	*E_int_(BSSE)*	*r*(Tt···X)	*E_int_*	*E_int_(BSSE)*	*r*(Tt···X)	*E_int_*	*E_int_(BSSE)*	*r*(Tt···X)	*E_int_*	*E_int_(BSSE)*	*r*(Tt···X) ^b^
[I_4_C···F^−^]	−27.36	−19.72	2.690	−19.23	−19.2	2.744	−20.11	−18.7	2.663	−26.00	−16.35	2.665
[I_4_C···Cl^−^]	−10.97	−8.10	3.671	−7.87	−7.84	3.787	−11.08	−10.07	3.496	−9.99	−5.93	3.665
[I_4_C···Br^−^]	−8.12	−6.61	4.001	−6.65	−6.61	4.097	−10.13	−8.93	3.710	−7.25	−4.64	3.996
[I_4_C···I^−^]	−6.02	−5.43	4.422	−5.60	−5.57	4.494	−9.25	−7.87	3.990	−4.80	−3.38	4.454
[I_4_C···At^−^]	−5.98	−5.48	4.558	−5.59	−5.57	4.603	−10.08	−7.95	4.009	−4.99	−3.20	4.506
[I_4_Si···F^−^]	−115.85	−115.48	1.643	−116.22	−116.16	1.638	−119.11	−116.45	1.639	−116.81	−112.15	1.637
[I_4_Si···Cl^−^]	−67.28	−66.93	2.191	−66.95	−66.88	2.190	−73.40	−71.00	2.177	−69.23	−64.99	2.192
[I_4_Si···Br^−^]	−9.75	−9.61	3.875	−9.85	−9.80	3.875	−62.88	−60.00	2.380	−58.1	−53.16	2.408
[I_4_Si···I^−^]	−7.42	−7.36	4.398	−7.52	−7.49	4.398	−53.22	−49.92	2.646	−8.51	−6.60	4.287
[I_4_Si···At^−^]	−7.13	−7.08	4.542	−7.23	−7.21	4.539	−13.09	−10.87	3.862	−8.44	−6.13	4.397
[I_4_Ge···F^−^]	−96.99	−96.53	1.797	−97.3	−97.17	1.792	−98.42	−95.4	1.787	−98.24	−93.43	1.789
[I_4_Ge···Cl^−^]	−60.16	−59.75	2.305	−59.77	−59.66	2.305	−65.32	−62.64	2.279	−62.93	−58.36	2.300
[I_4_Ge···Br^−^]	−51.15	−50.74	2.505	−50.93	−50.8	2.505	−58.29	−55.28	2.456	−54.44	−49.32	2.497
[I_4_Ge···I^−^]	−8.86	−8.79	4.233	−8.98	−8.94	4.233	−51.17	−47.91	2.703	−10.46	−8.28	4.049
[I_4_Ge···At^−^]	−8.34	−8.28	4.401	−8.44	−8.41	4.401	−51.73	−47.2	2.787	−9.95	−7.42	4.203
[I_4_Sn···F^−^]	−108.88	−99.20	1.965	−97.22	−97.14	1.966	−98.18	−95.5	1.955	−109.92	−96.48	1.950
[I_4_Sn···Cl^−^]	−72.20	−67.68	2.442	−65.22	−65.14	2.447	−70.39	−67.92	2.417	−74.18	−65.88	2.427
[I_4_Sn···Br^−^]	−61.88	−59.33	2.751	−58.32	−58.21	2.625	−65.06	−62.14	2.576	−63.91	−57.29	2.605
[I_4_Sn···I^−^]	−52.78	−51.71	2.873	−51.46	−51.38	2.869	−59.74	−56.43	2.792	−54.65	−49.58	2.847
[I_4_Sn···At^−^]	−51.94	−51.05	2.967	−50.56	−50.50	2.966	−60.82	−56.21	2.866	−55.43	−49.34	2.928
[I_4_Pb···F^−^]	−98.33	−88.98	2.075	−86.54	−86.48	2.078	−84.82	−81.43	2.063	−97.33	−84.05	2.057
[I_4_Pb···Cl^−^]	−67.00	−62.66	2.541	−59.88	−59.81	2.548	−63.4	−60.33	2.506	−67.71	−59.33	2.521
[I_4_Pb···Br^−^]	−58.51	−56.07	2.715	−54.77	−54.67	2.716	−59.91	−56.49	2.655	−59.42	−52.72	2.690
[I_4_Pb···I^−^]	−51.42	−50.43	2.950	−49.89	−49.83	2.948	−57.06	−53.30	2.854	−52.54	−47.36	2.916
[I_4_Pb···At^−^]	−51.74	−50.91	3.034	−50.20	−50.15	3.032	−59.42	−54.41	2.919	−54.51	−48.30	2.984

^a^ Interaction energies (*E_int_* and *E_int_(BSSE)*) and intermolecular distances (*r*) are in kcal mol^−1^ and Å, respectively. ^b^ Bond distances were obtained with [CCSD/def2-TZVPPD].

## Data Availability

This research did not report any data.
